# Multicentric study on malignant pleural mesothelioma and non-occupational exposure to asbestos

**DOI:** 10.1054/bjoc.2000.1161

**Published:** 2000-06-02

**Authors:** C Magnani, A Agudo, C A González, A Andrion, A Calleja, E Chellini, P Dalmasso, A Escolar, S Hernandez, C Ivaldi, D Mirabelli, J Ramirez, D Turuguet, M Usel, B Terracini

**Affiliations:** Cancer Epidemiology Unit, S Giovanni B Hospital and Regional Centre for Cancer Epidemiology and Prevention, Torino, Italy; Department of Epidemiology and Cancer Registration, Catalan Institute of Oncology (ICO), Av. Gran Via s/n, L'Hospitalet de Llobregat, Km 2.7, E-08907, Spain; Department of Pathology, Ospedale Martini, Torino, Italy; Centre de Seguretat i Condicions de Salut en el Treball (CSCST), Barcelona, Spain; Epidemiology Unit, Center for Study and Prevention of Cancer Firenze (CSPO), AO Careggi, Firenze, Italy; Department of Preventive Medicine and Public Health, Hospital Universitario Puerta del Mar, Cádiz, Spain; Agency for Environmental Protection (ARPA) Piemonte, Torino, Italy; Department of Pathology, Hospital Clinic, Barcelona, Spain; Former Documentation Services, Instituto Nacional de Seguridad e Higiene en el Trabajo and Centro de Investigación y Desarrollo de Barcelona (CSIC), Barcelona, Spain; Geneva Medical Inspectorate of Factories (OCIRT) and Geneva Cancer Registry, Geneva, Switzerland

**Keywords:** asbestos, environmental exposure, mesothelioma, case–control studies

## Abstract

Insufficient evidence exists on the risk of pleural mesothelioma from non-occupational exposure to asbestos. A population-based case–control study was carried out in six areas from Italy, Spain and Switzerland. Information was collected for 215 new histologically confirmed cases and 448 controls. A panel of industrial hygienists assessed asbestos exposure separately for occupational, domestic and environmental sources. Classification of domestic and environmental exposure was based on a complete residential history, presence and use of asbestos at home, asbestos industrial activities in the surrounding area, and their distance from the dwelling. In 53 cases and 232 controls without evidence of occupational exposure to asbestos, moderate or high probability of domestic exposure was associated with an increased risk adjusted by age and sex: odds ratio (OR) 4.81, 95% confidence interval (CI) 1.8–13.1. This corresponds to three situations: cleaning asbestos-contaminated clothes, handling asbestos material and presence of asbestos material susceptible to damage. The estimated OR for high probability of environmental exposure (living within 2000 m of asbestos mines, asbestos cement plants, asbestos textiles, shipyards, or brakes factories) was 11.5 (95% CI 3.5–38.2). Living between 2000 and 5000 m from asbestos industries or within 500 m of industries using asbestos could also be associated with an increased risk. A dose–response pattern appeared with intensity of both sources of exposure. It is suggested that low-dose exposure to asbestos at home or in the general environment carries a measurable risk of malignant pleural mesothelioma. © 2000 Cancer Research Campaign


British Journal of Cancer (2000) 83(1), 104–111
© 2000 Cancer Research Campaign
doi: 10.1054/ bjoc.2000.1161, available online at http://www.idealibrary.com on IDEAL®

### Multicentric study on malignant pleural mesothelioma and non-occupational exposure to asbestos

**C Magnani[1], A Agudo[2], CA González[2], A Andrion[3], A Calleja[4], E Chellini[5], P Dalmasso[1], A Escolar[6], S Hernandez[4], C Ivaldi[1], D Mirabelli[7], J Ramirez[8], D Turuguet[9], M Usel[10] and B Terracini[1]**

[1] Cancer Epidemiology Unit, S Giovanni B Hospital and Regional Centre for Cancer Epidemiology and Prevention, Torino, Italy; [2]Department of Epidemiology and Cancer Registration, Catalan Institute of Oncology (ICO), Av. Gran Via s/n, Km 2.7, E-08907, L'Hospitalet de Llobregat, Spain; [3]Department of Pathology, Ospedale Martini, Torino, Italy; [4]Centre de Seguretat i Condicions de Salut en el Treball (CSCST), Barcelona, Spain; [5]Epidemiology Unit, Center for Study and Prevention of Cancer Firenze (CSPO), AO Careggi, Firenze, Italy; [6]Department of Preventive Medicine and Public Health, Hospital Universitario Puerta del Mar, Cádiz, Spain; [7]Agency for Environmental Protection (ARPA) Piemonte, Torino, Italy; [8]Department of Pathology, Hospital Clinic, Barcelona, Spain; [9]Former Documentation Services, Instituto Nacional de Seguridad e Higiene en el Trabajo, and Centro de Investigación y Desarrollo de Barcelona (CSIC), Barcelona, Spain; [10]Geneva Medical Inspectorate of Factories (OCIRT) and Geneva Cancer Registry, Geneva, Switzerland

**Summary** Insufficient evidence exists on the risk of pleural mesothelioma from non-occupational exposure to asbestos. A population-based case–control study was carried out in six areas from Italy, Spain and Switzerland. Information was collected for 215 new histologically confirmed cases and 448 controls. A panel of industrial hygienists assessed asbestos exposure separately for occupational, domestic and environmental sources. Classification of domestic and environmental exposure was based on a complete residential history, presence and use of asbestos at home, asbestos industrial activities in the surrounding area, and their distance from the dwelling. In 53 cases and 232 controls without evidence of occupational exposure to asbestos, moderate or high probability of domestic exposure was associated with an increased risk adjusted by age and sex: odds ratio (OR) 4.81, 95% confidence interval (CI) 1.8–13.1. This corresponds to three situations: cleaning asbestos-contaminated clothes, handling asbestos material and presence of asbestos material susceptible to damage. The estimated OR for high probability of environmental exposure (living within 2000 m of asbestos mines, asbestos cement plants, asbestos textiles, shipyards, or brakes factories) was 11.5 (95% CI 3.5–38.2). Living between 2000 and 5000 m from asbestos industries or within 500 m of industries using asbestos could also be associated with an increased risk. A dose–response pattern appeared with intensity of both sources of exposure. It is suggested that low-dose exposure to asbestos at home or in the general environment carries a measurable risk of malignant pleural mesothelioma. © 2000 Cancer Research Campaign

**Keywords:** asbestos; environmental exposure; mesothelioma; case–control studies

There is convincing evidence that pleural malignant mesothelioma is associated with occupational exposure to all commercial forms of asbestos (Landrigan, 1998; WHO, 1998). Although most cases of mesothelioma show a definite history of asbestos exposure at work, in population studies there is a proportion of cases that do not report any occupational exposure throughout their working life. Therefore, attention has turned to the potential risk associated with exposure at the lower doses in the general environment (Landrigan 1998).

Two circumstances for possible non-occupational exposure to asbestos have been investigated: domestic and environmental exposure. The former results from asbestos fibres brought home by workers exposed in the workplace (Gardner and Saracci, 1989). Environmental exposure may result from residence in the vicinity of asbestos mines, mills, or factories using asbestos. In many studies there is a single well-identified source of asbestos pollution termed a 'neighbourhood exposure'. Another kind is due to residence in areas where the soil is naturally rich in asbestos or similar fibres. Both sets of circumstances have led to localized outbreaks of pleural mesotheliomas, large enough to be first recognized in the absence of formal epidemiological studies (Gardner and Saracci, 1989). The latter are needed, however, to investigate whether the industrial use of asbestos may produce sufficient environmental pollution to cause asbestos-related disease. Rarely, mesotheliomas may occur in recognizable geographical or temporal clusters when the exposure is relatively high, but they will go unnoticed when exposure is low. Although asbestos is widely found in the environment, insufficient evidence exists on the risk of mesothelioma as a consequence of general environmental exposure (Siemiatycki and Boffeta, 1998). The extent to which the general population is exposed and the potential effects of such low-dose exposure are a matter of controversy.

A multicentric population-based case–control study was therefore carried out with the main aim of measuring risk associated with low-intensity, non-occupational exposure to asbestos.

*Received 26 October 1999*
*Revised 21 January 2000*
*Accepted 17 February 2000*

*Correspondence to:* A Agudo

### MATERIALS AND METHODS

The study was carried out in six areas in three European countries: the metropolitan area of the city of Torino (population 1.3 million), and the 13 towns included in the Local Health Authority of Casale Monferrato (100 000 inhabitants) in Piedmont, as well as the provinces of Firenze and Prato (population 1.2 million) in Italy; the provinces of Barcelona and Cádiz (population 4.6 and 1.1 million respectively) in Spain; and the Canton of Genève (400 000 inhabitants) in Switzerland.

Residents in the study areas with newly diagnosed primary malignant pleural mesothelioma between 1 January 1995 and 31 December 1996 were potentially eligible cases, except in Barcelona where the study included also cases diagnosed in 1993 and 1994, and in Torino where the recruitment ended in April 1997. All areas are covered by population cancer or mesothelioma registries except the two provinces of Barcelona and Cádiz. A surveillance system based on pathology departments in all the hospitals in the study areas was set up. All cases included were histologically confirmed, according to specific criteria defined by a panel of pathologists. An independent pathologist in each country reviewed diagnostic slides and a review panel was organized twice for the evaluation of dubious cases and 20% of all cases randomly selected. Agreement in this sample was close to 100%.

Controls were selected as a random sample from the population in Italian centres and Geneva. In the Spanish centres controls were randomly selected from patients discharged from all hospitals in the area, excluding those with asbestos-related conditions as described elsewhere (Agudo and González, 1999) which minimized the effect of the catchment area of the hospital. This procedure was adopted to avoid the low participation found in a population sample during the pilot study. The control group was selected according to the age–sex structure expected for cases (frequency matching) with a sample size twice the number of cases.

Cases and controls were interviewed at home or at the hospital by trained interviewers. However, when the subject had died, a relative provided the information. Almost all controls (98%) were directly interviewed, while a proxy respondent was needed for one-third of cases (Table 1). Interviews lasted on average 66 min for cases and 52 min for controls. The questionnaire included demographic characteristics, smoking habits, radiation treatment, lifelong occupational history with specific sections for 33 industrial activities and occupations with possible asbestos use, occupations held by spouse, parents and other cohabitants (with additional details for asbestos-related occupations) and lifelong residential history, including address and description of dwellings and their neighbourhood environment.

Lifetime asbestos exposure was assessed from questionnaire data by a panel of industrial hygienists, together with their knowledge of asbestos use in the study areas (Appendix 1). Standardized criteria were followed to assess the probability and intensity of asbestos exposure separately for occupational, domestic and environmental sources, blinded to the case–control condition of the subject. The classification of domestic and environmental exposure was based on the residential history. For each residence we recorded the dwelling characteristics, heating and air conditioning systems, insulation and other asbestos uses, as well as any cohabitants working in jobs with potential exposure to asbestos bringing

**Table 1** Main characteristics of total cases and controls participating in the study and cases and controls without occupational exposure to asbestos

| | Total | | Without occupational exposure | |
|---|---|---|---|---|
| | Cases $n$ = 215 (%) | Controls $n$ = 448 (%) | Cases $n$ = 53 (%) | Controls $n$ = 232 (%) |
| Centre | | | | |
| Casale | 23 (10.7) | 97 (21.7) | 14 (26.4) | 62 (26.7) |
| Turin | 41 (19.1) | 68 (15.2) | 8 (15.1) | 35 (15.1) |
| Florence | 15 (7.0) | 18 (4.0) | 1 (1.9) | 6 (2.6) |
| Barcelona | 117 (54.4) | 227 (50.7) | 28 (52.8) | 109 (47.0) |
| Cadiz | 15 (7.0) | 30 (6.7) | 2 (3.8) | 18 (7.8) |
| Geneva | 4 (1.9) | 8 (1.8) | – | 2 (0.9) |
| Gender | | | | |
| Male | 162 (75.3) | 322 (71.9) | 21 (39.6) | 130 (56.0) |
| Female | 53 (24.7) | 126 (28.1) | 32 (60.4) | 102 (44.0) |
| Age group | | | | |
| ≤ 44 years | 8 (3.7) | 29 (6.5) | 3 (5.7) | 16 (6.9) |
| 45–64 years | 78 (36.3) | 153 (34.2) | 23 (43.4) | 75 (32.3) |
| 65–74 years | 90 (41.9) | 182 (40.6) | 19 (35.8) | 89 (38.4) |
| ≥ 75 years | 39 (18.1) | 84 (18.7) | 8 (15.1) | 52 (22.4) |
| Education level[a] | | | | |
| Primary not completed | 53 (26.2) | 97 (22.7) | 14 (27.5) | 46 (20.7) |
| Primary completed | 68 (33.7) | 166 (38.8) | 14 (27.5) | 92 (41.4) |
| Secondary school | 44 (21.8) | 83 (19.4) | 11 (21.6) | 41 (18.5) |
| High school | 32 (15.8) | 57 (13.3) | 11 (21.6) | 29 (13.1) |
| University | 5 (2.5) | 25 (5.8) | 1 (2.0) | 14 (6.3) |
| Type of respondent | | | | |
| Subject | 145 (67.4) | 438 (97.8) | 38 (71.7) | 225 (97.0) |
| Spouse | 35 (16.3) | 4 (0.9) | 9 (17.0) | 3 (1.3) |
| Son/daughter | 31 (14.4) | 2 (0.4) | 5 (9.4) | 1 (0.4) |
| Other | 4 (1.9) | 4 (0.9) | 1 (1.9) | 3 (1.3) |

[a] For 13 cases (two without occupational exposure) and 20 control (ten without occupational exposure) information on education was missing. Percentages are calculated over 202 cases and 428 controls (total) and 51 cases and 222 controls (subgroup without occupational exposure).

*© 2000 Cancer Research Campaign* 

clothes home for cleaning. Evaluation of environmental exposure depended on the industrial activities in the surroundings and their distance from the subject's home (Marconi et al, 1989). Classification was independent of the time and duration of exposure. For each source separately the highest probability of exposure throughout all periods was considered as the subject's probability of asbestos exposure, while the highest intensity in periods used to assign probability was recorded as the subject's intensity. Duration was measured as the number of years between the start and the end of exposure in each period, and latency was measured as the length of time from onset of exposure to the date of diagnosis in cases and the date of interview in controls. Risk assessment associated with domestic and environmental exposure was carried out for subjects without occupational exposure. Potential exposure to asbestos at the workplace according to its probability and intensity was therefore assessed by industrial hygienists very carefully.

Relative risk was estimated by unconditional logistic regression (Breslow and Day, 1980). Odds ratios (OR) with corresponding 95% confidence intervals (CI) were calculated for each exposure category as compared to the never exposed (the reference category). Taking into account the stratified sampling design, all the estimates were adjusted by centre, sex and age. Certain analyses by study area were limited to the three largest centres (Casale, Torino and Barcelona).

### RESULTS

A total of 215 cases and 448 controls were included in the study (Table 1). Almost three-quarters of the cases were males, with a mean age of 65 years. Participation rates were 94% and 82% for cases and controls respectively, ranging from 72% for cases in Casale and 40% for controls in Geneva to 100% in Cádiz for both cases and controls. Overall 68.4% of cases and 43.5% of controls were classified as having had some degree of occupational exposure to asbestos, which was considered to be 'certain' for 39.1% of cases and 13.1% for controls. Age- and sex-adjusted OR and 95% CI were 1.6 (95% CI 0.9–2.9), 3.0 (95% CI 1.8–5.1) and 7.9 (95% CI 4.8–13.1) for 'low', 'middle or high', and 'certain' probability of occupational exposure respectively. Occupationally exposed cases and controls will not be considered further in the present context; analyses referring to domestic and environmental exposure are restricted to subjects who had never been occupationally exposed to asbestos.

For 53 cases and 232 controls the experts' panel found no evidence of occupational exposure to asbestos. Their distribution according to some variables is reported in Table 1. In this group, age distribution was very similar in cases and controls, but there was a striking predominance of females among cases and of males among controls.

The risks associated with domestic and environmental exposure to asbestos (mutually adjusted), separately for probability and intensity, are shown in Table 2. More than 30% of cases were classified as having a moderate or high probability of exposure to either source, while this proportion was lower than 10% for controls. For both sources and for both intensity and probability, ORs increased with increasing scores of exposure. Except for the 'low probability' or 'low intensity' categories, the increased risks were statistically significant being higher for environmental than for domestic exposure. A high risk (OR 11.5) was observed for high probability of environmental exposure, i.e. subjects who had lived at some time within 2000 m of a mine or asbestos works.

The environmental exposure to asbestos started at younger ages and lasted longer for cases than for controls: mean age at starting

**Table 2** Risk of pleural mesothelioma according to levels (see Appendix 1) of domestic and environmental exposure to asbestos

| | Cases *n* (%) | Controls *n* (%) | OR[a] | 95% CI |
|---|---|---|---|---|
| **(a) Probability** | | | | |
| *Domestic exposure* | | | | |
| Never exposed | 18 (34.0) | 146 (62.9) | 1 | – |
| Low probability | 14 (26.4) | 32 (13.8) | 2.05 | (0.83–5.09) |
| Middle or high probability | 16 (30.2) | 15 (6.5) | 4.81 | (1.77–13.1) |
| Unknown | 5 (9.4) | 39 (16.8) | 0.74 | (0.22–2.53) |
| *Environmental exposure* | | | | |
| No or background exposure | 20 (37.7) | 176 (75.9) | 1 | – |
| Low probability | 8 (15.1) | 20 (8.6) | 2.70 | (0.87–8.37) |
| High probability | 17 (32.1) | 21 (9.1) | 11.5 | (3.47–38.2) |
| Unknown | 8 (15.1) | 15 (6.5) | 3.54 | (1.20–10.4) |
| **(b) Intensity** | | | | |
| *Domestic exposure* | | | | |
| Never exposed | 18 (34.0) | 146 (62.9) | 1 | – |
| Low intensity | 15 (28.3) | 34 (14.7) | 2.01 | (0.84–5.06) |
| Middle intensity | 6 (11.3) | 7 (3.0) | 5.68 | (1.39–23.3) |
| High intensity | 9 (17.0) | 4 (1.7) | 7.83 | (1.69–36.2) |
| Unknown | 5 (9.4) | 41 (17.7) | 0.75 | (0.21–2.69) |
| *Environmental exposure* | | | | |
| No or background exposure | 20 (37.7) | 176 (75.9) | 1 | – |
| Low intensity | 6 (11.3) | 19 (8.2) | 2.23 | (0.65–7.64) |
| Middle intensity | 13 (24.5) | 19 (8.2) | 9.48 | (2.46–36.5) |
| High intensity | 6 (11.3) | 3 (1.3) | 45.0 | (6.38–318.0) |
| Unknown | 8 (15.1) | 15 (6.5) | 3.42 | (1.15–10.2) |

[a] ORs adjusted by centre, sex and age; effects of the two sources of exposure (domestic and environmental) are mutually adjusted as well.

 *© 2000 Cancer Research Campaign*

**Table 3** Risk of pleural mesothelioma according to combined domestic and environmental exposure to asbestos, excluding those with unknown exposure to either source

| **Source of exposure** | **Probability of exposure to asbestos** | | | | |
|---|---|---|---|---|---|
| **Domestic**<br>**Environmental** | **No exposure**<br>**No background** | **Yes**<br>**No** | **No**<br>**Yes** | **Yes[a]**<br>**Yes[a]** | **High**<br>**High** |
| Cases (*n* = 41) | 9 | 11 | 7 | 8 | 6 |
| Controls (*n* = 182) | 128 | 27 | 11 | 11 | 5 |
| OR[b] | 1 | 4.92 | 11.5 | 9.53 | 21.9 |
| 95% CI | – | (1.78–13.6) | (2.83–46.5) | (2.88–31.5) | (4.21–114.1) |

[a] Any combination of domestic and environmental exposure excluding high/high; this category includes: 9 low/low, 4 low/high, 4 middle/low, 2 high/low. See Appendix 1 for the meaning of exposure categories. [b]ORs adjusted by centre, sex and age. Subjects never exposed to asbestos from any source are the reference category in this analysis. Further details and circumstances of exposure of cases in this table are given in Appendix 2.

was 14 and 21 years respectively, while mean duration was 39 and 27 years. These differences were even more evident among those with high probability of exposure. The pattern was different regarding domestic exposure: only subjects with the highest level of exposure had a mean duration greater among cases, but no differences were observed either for age at starting or duration for all categories combined (results not shown).

For 12 cases and 50 controls there was not enough information to classify them by probability of either source or exposure. After excluding these subjects, the combined effect of domestic and environmental exposure to asbestos was assessed for the remaining 41 cases and 182 controls (Table 3). Both routes of exposure, either alone or combined with the other, showed an increased, significant risk. Risk seems to be higher for subjects with environmental exposure only than for domestic exposure only, being quite high, but imprecise (OR 21.9, 95% CI 4.2–114.1) for those with simultaneous exposure to both sources at the highest category.

### DISCUSSION

For both domestic and environmental exposures, a dose–response relation was observed with intensity of exposure. Relative risks for environmental exposure seemed higher than for domestic exposure, but were based on small numbers, and confidence intervals overlapped. Compared to previous population-based investigations in Western countries, an original feature of the present study is its focus on non-occupational exposure to asbestos. Indeed, our database, after exclusion of persons occupationally exposed, is one of the largest ever investigated. Some of the main findings in our study relate to the 32 cases with known domestic and/or environmental exposure without evident occupational exposure; further details for such cases are given in Appendix 2.

A high probability of environmental exposure, defined as living within 2000 m of an asbestos mine or works such as asbestos cement plants, asbestos textiles, shipyards, or brakes factories, entailed an almost 12-fold increase in risk (Table 2). Living between 2000 and 5000 m of asbestos industries or within 500 m of industries using asbestos products (low probability) was associated with an increased, but not statistically significant risk. The study was carried out in six areas, in two of which (Casale Monferrato and Barcelona) asbestos-cement plants have been active for a long time. Indeed, the study was not confined to the surroundings of these sources but covered geographic areas characterized by a variety of other industrial activities, with potential for environmental asbestos pollution. As previously shown (Magnani et al, 1993, 1995, 1997), risks are very high for the general population in Casale, where a large asbestos cement factory was active over many decades. Nevertheless, a previously unreported excess risk associated with non-occupational exposure to asbestos was also detected in Barcelona and Torino (number of cases contributed by other areas was too small). The analysis presented in Table 2 according to centres showed an OR associated to a high probability of environmental exposure of 14.7 (95% CI 2.2–33.1) in Casale, based on 11 exposed cases, of 6.5 (95% CI 0.3–129.0) in Torino, with two exposed cases, and 10.9 (95% CI 0.9–129.8) in Barcelona, based on three exposed cases. Regarding cases with known domestic or environmental exposure (Appendix 2), apart from eight cases exposed at home by asbestos contaminated clothes, a recognized serious hazard, 16 cases lived in the vicinity of an asbestos cement plant, shipyard or foundry: six in Casale, five in Torino and five in Barcelona. However, nine of these 16 also reported domestic exposure; a similar proportion was found among controls, where 16 out of 27 environmentally exposed also reported domestic exposure (Table 3). Thus substantial data on previously unsuspected neighbourhood risk arise from five cases from Torino and five from Barcelona. Our results suggest that incidence rate of pleural mesothelioma among people with non-occupational asbestos exposure could be around ten times higher within 2000 m of asbestos industries.

Thus, the present study provides formal epidemiological evidence that environmental asbestos exposure typical of industrial areas can increase the mesothelioma risk in non-occupationally exposed persons. Before the present study, such evidence was limited to dramatic but rare circumstances in areas polluted with asbestos or similar materials, either naturally, as in certain rural areas of Greece (Sakellariou et al, 1996), Turkey (Yazicioglu et al, 1980) and New Caledonia (Luce et al, 1994), or derived from industrial point sources. Best documented examples of the latter are the excesses of mesothelioma in people living around a crocidolite mine in Australia (Hansen et al, 1998), as well as in women living in chrysotile mining areas in Quebec, although occupational or domestic exposure cannot be totally ruled out (Camus et al, 1998; Case, 1998), and the asbestos-cement plant in Casale Monferrato. In the latter, a significant OR of 11.6 was estimated for those never engaged in the asbestos-cement plant living within 1000 m of the factory (Magnani et al, 1997). On the contrary, two earlier case–control studies did not find differences in the

*© 2000 Cancer Research Campaign* 

proportion of cases and controls living in the vicinity of chrysotile mines in the USA and Canada (McDonald and McDonald, 1980) or a friction material production plant in Connecticut (Teta et al, 1983). A third study in Yorkshire (UK) observed that environmental exposure contributed little to the risk of mesothelioma after exclusion of occupational and domestic exposure (Howel et al, 1997).

In the present study a fivefold increase in risk has been estimated for high or moderate probability of being exposed to asbestos at home (Table 2). This relative risk was higher in Barcelona (OR 8.1, 95% CI 1.3–49.5, six exposed cases) than in Casale (OR 1.6, 95% CI 0.2–10.9, five exposed cases) and Torino (OR 1.3, 95% CI 0.1–13.9, two exposed case). The risk has long been recognized and has been mainly attributed to exposure to fibres brought home with the clothes of asbestos workers (Vianna and Polan, 1978; McDonald and McDonald, 1980; Gardner and Saracci, 1989; Howel et al, 1997). The present study, however, suggests that exposure at home from handling asbestos material for maintenance and from presence of asbestos material susceptible to damage also increases risk. In a previous study in Casale a relative risk around 8 was estimated in a cohort of non-occupationally exposed wives of workers in the asbestos cement plant (Magnani et al, 1993). In a study in the USA the pulmonary asbestos concentrations among household contacts of asbestos workers were comparable to those found in occupationally exposed individuals (Roggli and Longo, 1991). After discarding exposure by washing clothes and neighbourhood exposure, for the remaining eight cases in Appendix 2, the only known source of exposure was the presence of some form of asbestos at home. Six of such cases, all in Barcelona, had an asbestos roof (one of them also reported asbestos in the electric heating) and two had other form of asbestos at home. It has been shown that weathered asbestos products may release fibres leading to concentrations from 0.2 to 1.2 fibres per litre in the environment (Spurny 1989). It is possible that levels in houses with asbestos roof in Barcelona are particularly high, but other explanations of our findings cannot be ruled out, such as another source of asbestos exposure for subjects living in such dwellings, or features of the design of the study, such as the use of hospital controls, or inaccuracy of the information provided by a relative. The lack of cases reporting asbestos roof in other areas in the study suggests that risk is negligible except perhaps in Barcelona, but could also reflect low statistical power.

Several potential sources of bias in the present study must be considered. Questionnaires were converted into levels of likelihood and intensity of exposure form occupational, domestic and environmental sources by a panel of experts, blinded to the status of the subject at issue. At least for environmental exposure, it may be inferred from the high OR for the ‘unknown’ category that the panel tended to be conservative in assigning a definite level of exposure. On the other hand, individuals with low education (more likely to have occupational exposure to asbestos) may have given inaccurate histories, thus spuriously increasing risk estimates for domestic and environmental sources. The context of the present study is unusual, since quality of classification of non-occupational exposure is influenced by the quality of occupational exposure. The possibility of overestimating ORs associated with domestic or environmental exposure cannot be ruled out.

Incomplete information (and thus erroneous allocation of a subject to a particular exposure category) as long as this is randomly distributed among cases and controls, will cause non-differential misclassification, shifting the risk estimate to the null in dichotomous exposures. In the case of polytomous exposure measures (Dosemeci et al, 1990) misclassification mainly produces an incorrect estimate of the slope of dose–response.

A major concern is the low quality of information provided by proxy respondents. The relatively high proportion of cases with proxies may have led to an artificially low proportion of cases with domestic or environmental exposure to asbestos and thus to an underestimation of risk. It might also have underestimated opportunities for occupational exposure, leading to the erroneous inclusion of occupationally exposed cases in our analysis. Furthermore, cases (and perhaps relatives of deceased cases) being aware of the hypothesis studied, may recall better than controls, thus producing overestimation of risk. Within the present investigation, a validation study was carried out in 18 cases from Barcelona: subjects provided direct information and, after they died, a proxy was asked to answer the same questionnaire. Regarding classification of occupational exposure, the overall agreement measured by the index kappa was 0.59, and it increased to 0.79 when only answers from the spouse were considered. For these subjects direct interviews lasted 55 min vs 71 min for proxies, and the average of different jobs reported by index subjects was 5.1 (ranging from 2 to 15) and 5.2 (ranging from 1 to 12) by proxies. Finally (and most relevant) the classification of subjects by the panel of experts did not change using either sources of information.

Pleural mesothelioma is known to be asbestos-related, which may lead to non-random misdiagnosis favouring inclusion of occupationally exposed cases. A diagnostic bias (Siemiatycki and Boffeta, 1998), however, is unlikely to have occurred in our study because of the inclusion of cases only after histological confirmation, and the revision by expert pathologists and/or a panel. Furthermore, non-random misdiagnosis driven by awareness of the occupational history would be limited to cases exposed in the workplace, which were excluded from the present analyses.

In conclusion, the results of this pioneering study confirm neighbourhood risk in Casale Monferrato and are suggestive of corresponding risk in Barcelona and Torino. An original observation is the association of mesothelioma with asbestos roofing in Barcelona. This requires confirmation in Barcelona itself as well as in other cities. It could be desirable to assess the problem by directly estimating local rates: unfortunately, this is often unfeasible mainly because denominators are not available. Indeed, in the case of rare events with long latent periods, when approaching the possible association with environmental exposures, it is difficult to use a study design alternative to the case–control approach. Overall, our results suggest that non-occupational exposure to relatively low–doses of asbestos is a hazard that may contribute to the burden of mesothelioma over the next few decades (Peto et al, 1999).

### ACKNOWLEDGEMENTS

The study was funded by the BIOMED-I Programme (contract 93-1297), the Health Research Fund (FIS) of the Spanish Ministry of Health (contract 94-0550), the Italian Association for Research on Cancer and the Piemonte Region. The authors wish to thank the several collaborators of the study in different centres: Barcelona: Rafael Panadès, María J Bleda (field-work coordinator), and Cristina Mas (secretary); Cádiz: Manuel Beltrán (pathologist) and

© 2000 Cancer Research Campaign

José González-Moya (pneumologist), Piedmont: Mario Botta (oncologist), Pier Angelo Piccolini (pneumologist), Pier Giacomo Betta, Giovanni Bussolatti and Luciano Gubetta (pathologists), Giuliano Maggi (thoracic surgeon) and their collaborators, and Monica Garbero (secretary); Florence: Stefano Silvestri (industrial hygienist), Sergio Dini (pathologist) and Giusseppe Gorini (doctor). A special thanks to the interviewers: Marcella Democrito (Turin), Emilia Ferretti (Casale), Mercè Roca (Barcelona), Valentina Cacciarini (Florence). The 'working group' that participated in the design of the study and of the questionnaire also included: Tom Bellander (Florence), Etienne Guberan (Switzerland), Elsie Bonnyns and Daniel Roosels (Belgium); Niels Plato and Gunnar Hillerdal (Sweden), Robert van den Oever (Brussels), Elsebeth Lynge and Edith Raffne (Belgium), Athena Linos (Greece). The panel of pathologists included: Franco Mollo and Alberto Andrion (Turin), PierGiacomo Betta (Casale), Sergio Dini (Florence), Josep Ramirez (Barcelona), EK Verbeken (Leuven, Belgium), Anders Hjerpe (Huddinge, Sweden), KB Andersen (Herlev, Denmark).

### REFERENCES

Agudo A and González CA (1999) Secondary matching: a method for selecting controls in case–control studies on environmental risk factors. *Int J Epidemiol* **28**: 1130–1133

Breslow EN and Day N (1980) *Statistical Methods in Cancer Research. Vol 1. The Analysis of Case–control Studies*. IARC Scientific Publications No. 32. International Agency for Research on Cancer: Lyon

Camus M, Siemiatycki J and Meek B (1998) Nonoccupational exposure to chrysotile asbestos and the risk of lung cancer. *N Eng J Med* **338**: 1565–1571

Case BW (1998) Non-occupational exposure to chrysotile asbestos and the risk of lung cancer [letter]. *N Eng J Med* **339**: 1001

Dosemeci M, Wacholder S and Lubin JH (1990) Does nondifferential misclassification of exposure always bias a true effect toward the null value? *Am J Epidemiol* **132**: 746–748

Gardner MJ and Saracci R (1989) Effects on health of non-occupational exposure to airborne mineral fibres. In: *Non-Occupational Exposure to Mineral Fibres*, Bignon J, Peto J and Saracci R (eds), pp 375–397. IARC Scientific Publications No. 90. International Agency for Research on Cancer: Lyon

Hansen J, de Klerk NH, Musk AW and Hobbs MST (1998) Environmental exposure to crocidolite and mesothelioma. *Am J Respir Crit Care Med* **157**: 69–75

Howel D, Arblaster L, Swinburne L, Schweiger M, Renvoize E and Hatton P (1997) Routes of asbestos exposure and the development of mesothelioma in an English region. *Occup Environ Med* **54**: 403–409

Landrigan PJ (1998) Asbestos: still a carcinogen. *N Engl J Med* **338**: 1619–1620

Luce D, Brochard P, Quenel P, Salomon-Nekiriai C, Goldberg P, Billon-Galland MA and Goldberg M (1994) Malignant pleural mesothelioma associated with exposure to tremolite. *Lancet* **344**: 1777

McDonald AD and McDonald JC (1980) Malignant mesothelioma in North America. *Cancer* **146**: 1650–1656

Magnani C, Terracini B, Ivaldi C, Botta M, Budel P, Mancini A and Zanetti R (1993) Cohort study on mortality among wives of workers in the asbestos cement industry in Casale Monferrato, Italy. *Br J Ind Med* **50**: 779–784

Magnani C, Terracini B, Ivaldi C, Botta M, Mancini A and Andrion A (1995) Pleural malignant mesothelioma and non-occupational exposure to asbestos in Casale Monferrato, Italy. *Occup Environ Med* **52**: 362–367

Magnani C, Ivaldi C, Botta M and Terracini B (1997) Pleural malignant mesothelioma and environmental asbestos exposure in Casale Monferrato, Piedmont. Preliminary analysis of a case-control study. *Med Lav* **88**: 302–309

Marconi A, Cecchetti G and Barbieri M (1989) Airborne mineral fibre concentrations in an urban area near an asbestos-cement plant. In: *Non-Occupational Exposure to Mineral Fibres*, Bignon J, Peto J and Saracci R (eds), pp. 336–346. IARC Scientific Publications No. 90. International Agency for Research on Cancer: Lyon

Peto J, Decarli A, La Vecchia C, Levi F and Negri E (1990) The European mesothelioma epidemic. *Br J Cancer* **79**: 666–672

Roggli VL and Longo WE (1991) Mineral fibre content of lung tissue in patients with environmental exposures: household contacts vs building occupants. *Ann NY Acad Sci* **643**: 511–518

Sakellariou K, Malamou-Mitsi V, Haritou A, Koumpaniou C, Stachouli C, Dimoliatis ID and Constantopoulos SH (1996) Malignant pleural mesothelioma from non-occupational asbestos exposure in Metsovo (north-west Greece): slow end of an epidemic? *Eur Respir J* **9**: 1206–1210

Siemiatycki J and Boffetta P (1998) Invited commentary: is it possible to investigate the quantitative relation between asbestos and mesothelioma in a community-based study? *Am J Epidemiol* **148**: 143–147

Siemiatycki J, Nadon L, Lakhani R, Bégin D and Gérin M (1991) Exposure assessment. In: *Risk Factors for Cancer in the Workplace*, Siemiatycki J (ed), pp. 46–103. CRC Press: Boca Raton

Spurny KR (1989) Asbestos fibre release by corroded and weathered asbestos-cement products. In: *Non-Occupational Exposure to Mineral Fibres*, Bignon J, Peto J and Saracci R (eds), pp. 367–371. IARC Scientific Publications No. 90. International Agency for Research on Cancer: Lyon

Teta MJ, Lewinsohn HC, Meigs JW, Vidone RA, Mowad LZ and Flannery JT (1983) Mesothelioma in Connecticut, 1955–1977. Occupational and geographic associations. *J Occup Med* **25**: 749–756

Vianna NJ and Polan AK (1978) Non-occupational exposure to asbestos and malignant mesothelioma in females. *Lancet* **1**: 1061–103

WHO (1998) *Chrysotile Asbestos. Environmental Health Criteria. International Programme on Chemical Safety* (IPCS). The WHO Environmental Health Criteria No. 203. World Health Organization: Geneva

Yazicioglu S, Ilcayto R, Balci K, Sayli BS and Yorulmaz B (1980) Pleural calcification, pleural mesotheliomas, and bronchial cancers caused by tremolite dust. *Thorax* **35**: 564–569

*© 2000 Cancer Research Campaign*

**Appendix 1** Definition of categories of probability and intensity for domestic and environmental exposure to asbestos

| | Category | Description |
|---|---|---|
| **(a) Domestic exposure** | | |
| Probability | High (certain) | Relative employed in asbestos industry, working clothes brought home. Crushed asbestos cement in the garden/courtyard. Use of asbestos materials for work and maintenance at home. |
| | Middle (probable) | Presence of weathered asbestos material, or susceptible to damage and release of fibres with use (i.e. insulation material, gloves, ironing board) |
| | Low (possible) | Presence of asbestos material, unlikely to be damaged or to disperse fibres (e.g. in electric heating or hairdryer, asbestos roof) |
| | No exposure | Absence of asbestos material at home |
| | Unknown | Lack of information to determine presence or absence of asbestos material at home |
| Intensity | High | Subject handling asbestos at home or cleaning clothes of asbestos-exposed workers |
| | Middle | Passive exposure: asbestos material handled or asbestos contaminated clothes cleaned at home by cohabitants but not by the subject |
| | Low | Presence of asbestos material at home, not handled |
| | No exposure | Absence of asbestos material at home |
| | Unknown | Lack of information to determine presence or absence of asbestos material at home |
| **(b) Environmental exposure** | | |
| Probability | High (certain) | Asbestos mines or industries distant from home less than 2000 m (mines, asbestos cement, asbestos textiles, brakes and clutches lining, shipyards) |
| | Low (probable) | Asbestos mines or industries located between 2000 and 5000 m from home. Industries using asbestos less than 500 m from home (steel foundries, power plants, major chemical or petrochemical plants, major yards). |
| | No, background level | All other circumstances or conditions |
| | Unknown | Lack of information to determine environmental asbestos exposure |
| Intensity | High | Asbestos mines or industries less than 500 m from home. |
| | Middle | Asbestos mines or industries within 500–2000 m from home. |
| | Low | Asbestos mines or asbestos industries within 2000–5000 m from home. Industries using asbestos less than 500 m from home. |
| | No, background level | All other circumstances or conditions. |
| | Unknown | Lack of information to determine environmental asbestos exposure |

*© 2000 Cancer Research Campaign*

**Appendix 2** Description of 32 cases according to domestic and/or environmental exposure, excluded non-exposed subjects and those with unknown exposure to either source

| Case number | Centre | Diagnosis | Sex | Age | Domestic exposure Probability | Domestic exposure Description | Environmental exposure Probability | Environmental exposure Description |
|---|---|---|---|---|---|---|---|---|
| 100001 | Casale | 1995 | Female | 47 | – | | High | Asbestos cement plant distant from home less than 2000 m[a] |
| 100003 | Casale | 1995 | Male | 68 | – | | High | Asbestos cement plant distant from home less than 2000 m[a] |
| 100006 | Casale | 1995 | Male | 58 | High | Crushed asbestos material in the courtyard and use of asbestos material at home | High | Asbestos cement plant distant from home less than 2000 m[a] |
| 100007 | Casale | 1995 | Male | 61 | – | | Low | Asbestos cement plant distant from home less than 5000 m[a] |
| 100014 | Casale | 1996 | Female | 55 | – | | High | Asbestos cement plant distant from home less than 2000 m[a] |
| 100016 | Casale | 1995 | Female | 64 | High | Working clothes brought home by relative employed in asbestos industry | High | Asbestos cement plant distant from home less than 2000 m[a] |
| 100019 | Casale | 1995 | Female | 53 | High | Working clothes brought home by relative employed in asbestos industry | High | Asbestos cement plant distant from home less than 2000 m[a] |
| 100020 | Casale | 1995 | Female | 56 | High | Working clothes brought home by relative employed in asbestos industry | – | |
| 100151 | Casale | 1996 | Male | 53 | High | Crushed asbestos material in the courtyard | High | Asbestos cement plant distant from home less than 2000 m[a] |
| 201018 | Torino | 1995 | Male | 67 | Low | Asbestos material in the heating system | Low | Iron foundry beside home |
| 201020 | Torino | 1995 | Female | 78 | – | | High | Asbestos textile industry less than 2000 m from home |
| 201021 | Torino | 1995 | Female | 62 | High | Crushed asbestos material in the courtyard | Low | Chemical plant and iron foundry less than 500 m from home |
| 201024 | Torino | 1995 | Male | 55 | Low | Asbestos material in ventilating system | High | Asbestos textile industry less than 2000 m from home |
| 201241 | Torino | 1997 | Male | 70 | – | | Low | Iron foundry less than 500 m from home |
| 201264 | Torino | 1997 | Female | 60 | High | Working clothes brought home by relative employed in asbestos industry | – | |
| 300005 | Firenze | 1996 | Male | 69 | Middle | Asbestos material used in insulation (susceptible to damage) | – | |
| 405081 | Barcelona | 1995 | Female | 70 | High | Cleaning working clothes brought home by relative employed in industry dealing with insulation material in wagons | Low | Foundry distant from home less than 500 m |
| 405101 | Barcelona | 1995 | Female | 85 | Low | Asbestos roof | Low | Warehouse of construction material distant from home less than 500 m |
| 405151 | Barcelona | 1995 | Female | 68 | Low | Asbestos roof, asbestos in electric heating | – | |
| 405211 | Barcelona | 1995 | Male | 74 | Low | Asbestos roof, asbestos in electric heating | Low | Warehouse with asbestos material distant from home less than 500 m |
| 405271 | Barcelona | 1995 | Female | 55 | High | Cleaning working clothes brought home by relative employed in asbestos cement plant | High | Asbestos cement plant distant from home less than 2000 m[a] |
| 406011 | Barcelona | 1996 | Female | 65 | High | Crushed asbestos material in the courtyard | – | |
| 406071 | Barcelona | 1996 | Female | 87 | Low | Asbestos roof | – | |
| 406081 | Barcelona | 1996 | Male | 44 | Low | Asbestos roof | – | |
| 406131 | Barcelona | 1996 | Female | 74 | Low | Asbestos roof | – | |
| 490071 | Barcelona | 1993 | Female | 80 | Low | Asbestos roof | – | |
| 490301 | Barcelona | 1994 | Female | 35 | High | Use of asbestos material in work at home (installing asbestos roof) | – | |
| 490351 | Barcelona | 1994 | Female | 70 | – | | High | Shipyard distant from home less than 2000 m |
| 490441 | Barcelona | 1993 | Male | 45 | Low | Asbestos roof and pipes | High | Asbestos cement plant distant from home less than 2000 m[a] |
| 490461 | Barcelona | 1994 | Female | 88 | Low | Asbestos material in the heating system | Low | Foundry distant from home less than 500 m |
| 520021 | Cádiz | 1995 | Female | 83 | High | Cleaning working clothes brought home by relatives employed in a shipyard | High | Shipyard distant from home less than 2000 m |
| 520111 | Cádiz | 1996 | Female | 62 | High | Working clothes brought home by relative employed in a shipyard | – | |

[a] Indicates residence in a town/city where an asbestos cement plant is located.

© 2000 Cancer Research Campaign
